# Ethical challenges in integrating patient-care with clinical research in a resource-limited setting: perspectives from Papua New Guinea

**DOI:** 10.1186/1472-6939-14-29

**Published:** 2013-07-26

**Authors:** Moses Laman, William Pomat, Peter Siba, Inoni Betuela

**Affiliations:** 1 Papua New Guinea Institute of Medical Research, Madang, Madang Province, Papua New Guinea; 2 Papua New Guinea Institute of Medical Research, Goroka, Eastern Highlands Province, Papua New Guinea

**Keywords:** Developing countries, Resource-limited settings, Papua New Guinea, Ethical challenges, Therapeutic misconception, Inducement

## Abstract

**Background:**

In resource-limited settings where healthcare services are limited and poverty is common, it is difficult to ethically conduct clinical research without providing patient-care. Therefore, integration of patient-care with clinical research appears as an attractive way of conducting research while providing patient-care. In this article, we discuss the ethical implications of such approach with perspectives from Papua New Guinea.

**Discussion:**

Considering the difficulties of providing basic healthcare services in developing countries, it may be argued that integration of clinical research with patient-care is an effective, rational and ethical way of conducting research. However, blending patient-care with clinical research may increase the risk of subordinating patient-care in favour of scientific gains; therapeutic misconception and inappropriate inducement; and the risk of causing health system failures due to limited capacity in developing countries to sustain the level of healthcare services sponsored by the research. Nevertheless, these ethical and administrative implications can be minimised if patient-care takes precedence over research; the input of local ethics committees and institutions are considered; and funding agencies acknowledge their ethical obligation when sponsoring research in resource-limited settings.

**Summary:**

Although integration of patient-care with clinical research in developing countries appears as an attractive way of conducting research when resources are limited, careful planning and consideration on the ethical implications of such approach must be considered.

## Background

Developing countries carry a high proportion of the global disease burden and are increasingly becoming attractive sites for clinical research because of better funding in recent years [1]. On the contrary, research regulations in developed countries are becoming more restrictive, making research in the third world appear more attractive [2]. In view of this, the Council for International Organizations of Medical Sciences (CIOMS) in collaboration with the World Health Organization (WHO) developed the international ethical guidelines for biomedical research involving human subjects because of concerns with increasing research activities in third world countries [3]. The CIOMS guideline highlights ethical challenges with regards to the informed consent process, inappropriate inducement, vulnerable populations, post-trial access and therapeutic misconception. Although the guideline was developed to protect vulnerable populations in developing countries from exploitation, data on ethical issues affecting these settings are limited.

Therapeutic misconception is an example of an ethical challenge with limited data from a developing country’s perspective and refers to the tendency of participants in clinical research to confuse the conduct of research with medical care [4]. The term was originally used in randomised controlled clinical trials but is now widely used amongst researchers in various fields [5]. The confusion is particularly common when a clinician performing clinical research is also the patient’s healthcare provider [6]. Inducement is another example of an ethical challenge often encountered in resource-limited settings. In clinical research, appropriate inducement is considered acceptable if used as an incentive to improve the conduct of a study without coercion or harm to the patient and there is informed consent [3].

In developed countries where healthcare services are adequately funded, efficient and easily accessible, ethical challenges are less complex compared to developing countries. In developing countries, constant shortage of resources and failures in the healthcare system complicated by high rates of poverty and illiteracy often creates a more challenging situation [7-9]. In such settings, it may be argued that integrating patient-care with clinical research may be the most rational method of providing quality patient-care while consenting and recruiting patients. However, whether this approach of conducting research would be considered ethical or not is debatable depending on contextual differences between different settings and the nature of the research performed [10]. In this article, we specifically attempt to discuss ethical challenges often encountered during integration of clinical research with patient-care in developing countries, with perspectives from Papua New Guinea (PNG).

## Discussion

### Challenges in developing countries such as Papua New Guinea

Developing countries continue to bear a large proportion of the global disease burden with high morbidity and mortality rates [11]. Papua New Guinea is one such country in the Oceania region where 85% of its 7 million inhabitants reside in remote settings with limited healthcare services [12]. Its rugged mountainous terrains, dense rainforests, lowland swamps and remote atoll islands contribute to the inequalities and challenges of providing even the most basic healthcare service in PNG [12,13]. Complicated by deteriorating infrastructure and government services, late presentation to hospital often results in high maternal mortality rates [14]. Pneumonia, malaria, meningitis, malnutrition, sepsis and low birth weight are major causes of death in children <5 years of age [15]. Despite supplementary vaccination campaigns, immunization coverage for preventable fatal diseases like measles remain <70% and post-measles complications presenting as sub-acute sclerosing panencephalitis is considered to be the highest worldwide [16]. Four of the five known plasmodia species that cause malarial infections in humans co-exist in PNG [17]. *Streptococcus pneumoniae* and *Haemophilus influenzae* are leading causes of pneumonia and acute bacterial meningitis [18-20]. Although the *Haemophilus influenzae* type B vaccine was introduced in 2007, pneumococcal vaccine is not yet part of the vaccination schedule [21]. Unlike in African settings, the overall HIV prevalence in PNG is <1% [22], but tuberculosis is a major public health problem and the increasing incidence of multidrug-resistant tuberculosis continues to be a great concern [23]. Neglected tropical diseases such as yaws, helminthic infestations, scabies, lymphatic filariasis, trachoma, leprosy, cholera and dengue also contribute to a significant burden of morbidity and mortality in this population [24].

Considering these major challenges in providing basic healthcare services in a country such as PNG where the doctor to patient ratio is 0.49/10,000 [25], it would seem unethical for a researcher to turn away patients or reserve resources only for use in study patients when confronted with patients in desperate need of basic healthcare services. In such situations, integration of patient-care with clinical research appears as an effective, rational and ethical method of conducting research.

### Ethical implications of integrating patient-care with clinical research

i. The risk of subordinating patient-care in favour of scientific gain

Although integration of patient-care with clinical research appears attractive, there are important ethical implications that must be considered. Acceptance of this method of conducting research may lead to widespread exploitation of vulnerable people with limited options in developing countries particularly due to the temptation to subordinate the patient’s welfare to the objectives of the study. This is possible when the objectives of the research are extremely important with a high probability of improving the care of future patients [10]. In view of this risk, the declaration of Helsinki clearly states that the interest of science should never take precedence over the wellbeing of patients [26]. The Tuskegee syphilis study is a classic example of subordinating patient-care for the interest of science [27]. In that study, patients with syphilis were denied the best known treatment in their setting in favour of scientific gain. The justification provided by the investigators was that these patients would not have been treated if they were not in the study because of their low socioeconomic status.

In a similar instance, a randomised controlled trial in Uganda raised serious ethical concerns for observing HIV infected patients without treating them for over 30 months [28]. In addition, patients in that study found to have other sexually transmitted infections had to seek their own treatment, while HIV seronegative partners of HIV patients were not informed. Such examples highlight the fact that even with the most altruistic of motives, the risk of subordinating patient-care in favour of important research findings may become a real threat when integrating patient-care with clinical research in resource-limited settings.

ii. The risk of therapeutic misconception and inappropriate inducement

The risk of therapeutic misconception and inappropriate inducement in developing countries where illiteracy and poverty are common is also substantial. On the contrary, in well-resourced settings where healthcare services are well established and easily accessible, therapeutic misconception is perhaps easy to define since the role of a healthcare physician is distinctly different from the role of a research clinician. However, in resource-limited settings, often there is no clear distinction between a health service provider and a medical researcher and this often creates confusion, increasing the likelihood of therapeutic misconception. In addition, when clinical studies provide healthcare services, it may serve as an inappropriate inducement or coercion to potential participants particularly when patients are poor and such standard of healthcare services are limited or unavailable in their locality [29]. This will raise serious concerns regarding the ethical standards of the study.

iii. Challenges of sustaining health services funded by clinical studies

When clinical studies performed in resource-limited settings conform to the highest standard of care attainable in the local setting, the study is often considered as advantageous [29]. However, the challenge of sustainability comes after the research project ends. Because research projects often have limited lifespan and funding agencies do not provide funds for patient-care, the high standard set by the study would create undue stress on the health resources and infrastructure of the locality. In addition, the health system and workforce that have become dependent on the integrated research team and its resources will now have to perform without that support. Furthermore, since the expected roles and responsibilities of researchers collaborating with the health service providers are often not discussed explicitly and understood before the integration process at the start of the study, this often contributes to failures in the health system in resource-limited settings.

### Way forward in minimising ethical challenges

i. Importance of developing country ethics committees and institutions

The role of an ethics committee in a developing country in thoroughly reviewing research proposals is the most important step and often the only opportunity to determine the ethical and scientific aspects of a research proposal. This is mainly due to financial constrains in actively monitoring research projects, lack of experience (many ethics committees have existed for less than a decade as is the case in our setting), and lack of scientific expertise [30]. Nevertheless, ethics committees have played an important role in recent years in resource-limited settings in safeguarding the rights, safety and well-being of study participants.

Considering the ethical implications of integrating clinical research with patient-care, where possible, developing country ethics committees must find the balance between pushing for the highest standard of patient-care practically attainable in the host country to be given to study patients, while at the same time making sure the study does not harm the local healthcare system when sustainability is no longer possible at the completion of the study. This is of paramount importance since ethics committees in developed countries that approve clinical studies to be conducted in resource-limited settings in contrast, rarely understand the practicalities in resource-limited settings. Additionally, they do not have expertise in performing field research under challenging conditions and rarely understand the cultural issues involved in protecting study participants in resource-limited settings [30]. Therefore, without the input of local ethics committees, it would be difficult to contextualize and avoid ethical complications that may arise in resource-limited settings.

Furthermore, the research may find its greatest application only in developed countries but not in developing countries. For instance, a great deal of work on pneumonia research that has led to the development of the WHO guidelines for pneumonia in children and scientific data contributing to the development of pneumococcal vaccines in developed countries was pioneered in PNG since the 1960s [31]. Yet Papua New Guineans have not reaped the benefits of these research; pneumococcal vaccine is still unavailable in PNG after 50 years of research and pneumonia remains the leading cause of death in PNG children [32], the very reason that justified doing pneumonia research in PNG in the first place. Though this reflects many political and economic factors as well as the unethical trend of research in the past, these experiences have strengthened developing country research institutions such as the Papua New Guinea Institute of Medical Research and national ethics committees to ensure future research benefit both developing and developed countries.

ii. Community participation

In developed countries, autonomy is vital and a participant’s right is fundamental during the informed consent process [30]. However, in developing countries, community consent and participation as part of the informed consent process often precedes an individual informed consent process. For instance, in communities within PNG, without community consent it would be impossible to conduct research and misconceptions will be inevitable [33]. Complicated by high rates of illiteracy, whether a patient is formally educated enough to understand basics of a study concept in order to make an informed choose is challenging and further raises ethical questions. In a male dominated society, as is the case in many developing countries, often autonomy comes into conflict with beneficence, more commonly when a mother expects a male clinician or researcher to decide on her behalf. Consequently, community consent is an integral part of the informed consent process in developing country settings that must be emphasised.

iii. Minimising the risk of therapeutic misconception and inappropriate inducement

In a study investigating therapeutic misconception in Malawi, patients in a clinical trial rationally decided to participate because they wanted ‘better’ quality healthcare and not because of misconceptions [34]. This highlights the fact that even in settings where illiteracy is common, therapeutic misconceptions can be minimised if the informed consent process is facilitated by a competent person who understands the culture and language of the potential participant. However, this also highlights that when healthcare services are integrated with clinical research, the study may be perceived as a powerful inducement particularly if the standard of care provided by the clinical research is unavailable in the local setting.

Similarly, in PNG where the standards of care provided by well-funded research projects are often not comparable to poorly funded routine clinical care, patients usually enrol into clinical studies for what they think is ‘better’ clinical care. For instance, a simple full blood count examination often performed in patients in well-resourced settings as part of routine investigations is not routinely available even in some provincial hospitals in PNG. Continuous shortage of pharmaceuticals and infrastructure difficulties make simple healthcare interventions such as the role-out of HIV antiretroviral drugs and vaccinations impractical [35]. Consequently, the PNG paediatrics standard treatment guideline stipulates that *“It is everyone’s duty to ensure that all children are vaccinated. Every health facility and every health worker should ensure that children in their care are fully immunised*” [32]. This highlights the difficulties involved in providing basic healthcare services in PNG and the local guideline recommends opportunistic vaccination to be carried out by all health workers which includes medical researchers. Although critics may view this as inappropriate inducement, contextualized to the resource-limited setting; the provision of basic healthcare services while conducting research is an ethical obligation of all health workers mandated by local authorities. This stance is further supported by the Declaration of Helsinki which clearly states that lack of healthcare services should not justify not providing healthcare for patients than is generally available in a particular setting [26]. Provided there is valid consent, meaning that the participant is adequately informed, understands, and the informed consent is given by a competent person [36], the risk of therapeutic misconception and inappropriate inducement or coercion can be minimised.

iv. An ethical obligation of funding agencies

In studies conducted in resource-limited settings, apart from directly funding research activities, funding agencies are increasingly recognising the importance of capacity building in the host country. However, there is usually no funding allocated to the cost of providing basic healthcare services. From our perspectives as developing country researchers, it is difficult to ethically conduct research without providing basic healthcare services in such settings. Consequently, funding agencies must be aware of indirect costs that may include healthcare services that conform to the standard of care practically attainable in the host country [29]. This is an ethical responsibility that must be recognised by funding agencies and researchers who have an interest in conducting research in developing countries [10]. Although it is not their responsibility to provide healthcare services and study protocols often recommend that patients be referred to existing healthcare facilities, when accessibility and affordability of healthcare services are limited or non-existent, referring patients may mean subjecting them to sub-optimal patient-care that inevitably increases the likelihood of an adverse outcome. Therefore, study designs or limited resources in developing countries should not justify not providing basic healthcare services in such situations.

## Summary

Although integrating patient-care with clinical research in developing countries appears as an attractive method of conducting clinical research when resources are limited, careful planning and consideration on the ethical implications of such approach must be taken into consideration. This argument is not to suggest that vulnerable populations and poverty should justify a double ethical standard. Instead, we argue that ethical principles must be contextualised to the local setting, but a balance between proper patient-care conforming to the highest standard of healthcare practically attainable in a resource-limited setting must also be carefully weighed against the risk of ethical misconduct and harm to the local healthcare system. Ethical problems that may arise as a result of the integration process can be minimised if the ultimate allegiance of researchers is on patient-care; the input of local ethics committees and institutions are considered; and funding agencies acknowledge their ethical obligation when conducting research in resource-limited settings.

## Abbreviations

CIOMS: Council for International Organisations of Medical Sciences; WHO: World Health Organisation; PNG: Papua New Guinea; PNGIMR: Papua New Guinea Institute of Medical Research.

## Competing interests

The authors declare that they have no competing interests.

## Authors’ contributions

ML developed the arguments, reviewed the literature and wrote the first draft of the paper. ML, WP and IB critically revised the manuscript. IB, WP and PS edited the final manuscript. All authors approved the final version of the manuscript before submission.

## Pre-publication history

The pre-publication history for this paper can be accessed here:

http://www.biomedcentral.com/1472-6939/14/29/prepub
